# Re-imagining malaria: heterogeneity of human and mosquito behaviour in relation to residual malaria transmission in Cambodia

**DOI:** 10.1186/s12936-015-0689-0

**Published:** 2015-04-24

**Authors:** Charlotte Gryseels, Lies Durnez, René Gerrets, Sambunny Uk, Sokha Suon, Srun Set, Pisen Phoeuk, Vincent Sluydts, Somony Heng, Tho Sochantha, Marc Coosemans, Koen Peeters Grietens

**Affiliations:** Institute of Tropical Medicine, Antwerp, Belgium; AISSR, University of Amsterdam, Amsterdam, The Netherlands; National Center for Parasitology, Entomology and Malaria Control, Phnom Penh, Cambodia; University of Antwerp, Antwerp, Belgium; Partners for Applied Social Sciences (PASS) International, Tessenderlo, Belgium; School of International Health Development, Nagasaki University, Nagasaki, Japan

## Abstract

**Background:**

In certain regions in Southeast Asia, where malaria is reduced to forested regions populated by ethnic minorities dependent on slash-and-burn agriculture, malaria vector populations have developed a propensity to feed early and outdoors, limiting the effectiveness of long-lasting insecticide-treated nets (LLIN) and indoor residual spraying (IRS). The interplay between heterogeneous human, as well as mosquito behaviour, radically challenges malaria control in such residual transmission contexts. This study examines human behavioural patterns in relation to the vector behaviour.

**Methods:**

The anthropological research used a sequential mixed-methods study design in which quantitative survey research methods were used to complement findings from qualitative ethnographic research. The qualitative research existed of in-depth interviews and participant observation. For the entomological research, indoor and outdoor human landing collections were performed. All research was conducted in selected villages in Ratanakiri province, Cambodia.

**Results:**

Variability in human behaviour resulted in variable exposure to outdoor and early biting vectors: (i) indigenous people were found to commute between farms in the forest, where malaria exposure is higher, and village homes; (ii) the indoor/outdoor biting distinction was less clear in forest housing often completely or partly open to the outside; (iii) reported sleeping times varied according to the context of economic activities, impacting on the proportion of infections that could be accounted for by early or nighttime biting; (iv) protection by LLINs may not be as high as self-reported survey data indicate, as observations showed around 40% (non-treated) market net use while (v) unprotected evening resting and deep forest activities impacted further on the suboptimal use of LLINs.

**Conclusions:**

The heterogeneity of human behaviour and the variation of vector densities and biting behaviours may lead to a considerable proportion of exposure occurring during times that people are assumed to be protected by the distributed LLINs. Additional efforts in improving LLIN use during times when people are resting in the evening and during the night might still have an impact on further reducing malaria transmission in Cambodia.

## Background

The effectiveness of existing malaria control measures has led to new radical challenges in settings where malaria has successfully been reduced. Residual transmission, defined as persisting transmission after full coverage of long-lasting insectical nets (LLIN) or indoor residual spraying (IRS) has been achieved, challenges malaria elimination [1,2]. Standard control programmes and measures seldom consider human heterogeneity such as local sociocultural variability, examples of which are mobility [3], the lower than expected uptake of preventive measures in certain vulnerable populations [4,5] and difficulties achieving optimal adherence to anti-malarial treatment [6-8]. Low malaria transmission in pre-elimination contexts might additionally cause a decreasing perception of risk, potentially leading to a lower use of malaria preventive measures [9,10]. In addition, vector behaviour has proven to be equally heterogeneous, as it can adapt to and thus challenge standard vector control tools [1,11,12]. After the scaling-up of LLINs in Southeast Asia, remaining vector populations developed a propensity to predominantly feed early and/or outdoors, as well as rest outdoors after feeding, fundamentally limiting the effectiveness of LLIN and IRS [13,14].

These challenges invoke memories of the Global Malaria Eradication Programme embarked upon in 1955 [2,15,16]. Despite significant local successes, in many regions malaria resurged after the abandonment of control programmes, hitting hard on populations that had lost much of their immunity against the disease [16]. After the success of vector control with DDT, which transformed endemic areas into settings with a “manageable proportion” of infective cases– a situation comparable to the current low transmission context of Southeast Asia – interventions that successfully interrupted transmission were still lacking [15,16]. These failures strongly related to the application of a centrally defined and standardized plan to locally diverse epidemiological, entomological and socio-cultural contexts. The importance of this local heterogeneity, including social and cultural barriers and vector behaviour, which prevented success in the past, must not be forgotten as this is once again key to reaching the current goal of elimination [15-17].

Due to the changing malaria epidemiology, outdoor transmission is becoming an important focus for malaria control strategies today. In the Greater Mekong Region, malaria is now mainly reduced to forested regions, populated by ethnic minorities [4,9,11,18], where residual transmission is hypothesized to mainly occur by vectors that are active during early evening and morning hours when people are not sleeping in mosquito nets. In addition, the high diversity of potential vector species together with highly heterogeneous malaria vector behaviour both between and within vector species, complicates the malaria epidemiology [1,11]. The same vectors can behave differently in different villages in the same region, which is due to vector biology, ecology, human behaviour and the presence of vector control [12,13].

The evolving interplay of vector and human behaviour in Southeast Asia’s remaining transmission foci (i.e. forested areas) challenges elimination goals [19], echoing problems encountered during the first eradication campaign. Starting from the premise that, in order to target residual transmission in these low transmission settings, a clear understanding is needed of when and how humans and vectors meet, the focus of this research was on the sociocultural heterogeneity in Ratanakiri province, Cambodia, representing a transmission zone in a country otherwise well on its way towards a pre-elimination phase. This paper presents the results from an anthropological study and an entomological study, which were ancillary to an epidemiological trial investigating the effectiveness of the mass use of topical repellents in addition to the use of LLINs in controlling malaria infections.

## Methods

### Study site and population

#### Population

Cambodia is inhabited by approximately 90% ethnic Khmer. However, there is a small ethnic minority population located mostly in the northeast, in Ratanakiri and Mondulkiri provinces. These populations are part of a larger cultural area, which extends from Laos in the north through the central highlands of Vietnam in the east and finally Ratanakiri and Mondulkiri in the south [20]. The largest ethnic groups in Ratanakiri are the Jarai, the Tompuon and the Kreung, each with a distinct language and cultural system in terms of kinship and political organization [21]. A characteristic shared by these ethnic groups is that they usually combine slash-and-burn agriculture with hunting, fishing and gathering various forest products. As distances between farms in the forest, rice fields and villages can be substantial, many families maintain residences at each location, and move from one place to another according to the agricultural cycle and the forest farm or rice field’s requirements [8,21].

#### Malaria

Malaria transmission is perennial with two peaks, June-July and October-November, the rainy season lasting from May to October. At the end of the malaria season of 2012, the overall PCR prevalence in Ratanakiri, as recorded by the MalaResT study (cfr. Infra), was estimated at 4.9% [22]. Species-specific areas with elevated risk of infection have been detected for all *Plasmodium* species. The clusters for falciparum, vivax and ovale malaria appear in the north of the province along the main river, while the cluster for Malariae is situated in the south of the province [23]. The primary vectors in Ratanakiri are *Anopheles minimus* and *Anopheles dirus*, and many secondary vectors are present. These vectors are generally exophagic and exophilic, and their densities and behaviour vary extensively per village [13]. Early biting proportion (EBP), which is calculated as the biting activity before 22.00 (i.e. assumed human sleeping time), was observed to be around 50% in Ratanakiri in 2005, the proportion of infectious bites before 22.00 was 29% [13].

### Study context

This study took place in the framework of an intervention trial (MalaResT), which aimed to raise evidence on the effectiveness of the mass use of topical repellents in addition to LLINs to reduce malaria infections. In this intervention trial, 113 of the most endemic villages in Ratanakiri were randomly assigned to a control arm, in which every household received one LLIN per one person, or to an intervention arm, where in addition to LLINs topical repellents were distributed biweekly to every household one bottle per one person. For the epidemiological trial, sample size calculations were based on an expected outcome of 40% difference in malaria prevalence between intervention and control arm. During each of the four malariometric surveys organized in the trial, the aim was to collect blood samples of 65 randomly selected participants within each community. Blood samples were analysed using PCR detection in a mobile laboratory, allowing for a sensitive and rapid malaria diagnostic strategy in the field [22], alongside a small questionnaire on overnight stays at different locations in the month prior to the survey.

### Research strategy

The research used a sequential mixed methods study design in which qualitative ethnographic research and quantitative survey research methods were used to complement the qualitative findings (in standard annotation [qual - > quan]) [24]. Qualitative ethnographic data were collected in local communities to acquire an in-depth understanding of the study setting and population while a cross-sectional and a structured observation survey aimed at quantifying relevant variables from the qualitative study. In addition, a malariometric survey was performed, thus enabling to link previously determined relevant variables to malaria infection, and entomological surveys were performed in selected villages.

### Quantitative study

#### Data collection

The exploratory and in-depth ethnographic research was done in 2012 in a selection of villages included in repellent study mentioned above, more specifically in the intervention villages (with repellents and LLINs) of Kachon Kraom, Lom and Sayos in respectively Voen Sai, Oyadao and Lumphat district, as well as during shorter visits in other communities around these villages, including some control villages (with only LLINs).

Participant observation and in-depth interviewing were carried out within the qualitative strand of the study. Participant observation consisted of observations and reiterated informal conversations and was especially used as an exploratory technique to detect unforeseen variables and to contrast stated opinions with actual behaviour, as it constitutes a respondent independent data collection tool. In 2012, 153 in-depth interviews were recorded and transcribed.

Multiple purposive sampling techniques were used, where informants were selected in relation to emerging preliminary results. In order to increase confidentiality with respondents and consequent reliability of the data, snowball sampling techniques - participants introducing us to other participants - were also used.

### Quantitative study

#### Data collection

For the quantitative strand, two surveys were carried out. From August till November 2012, a cross-sectional survey was performed with a close-ended structured questionnaire based on relevant variables emerging from the ethnographic strand. It explored the following topics: mobility between farms, fields and villages, repellent use, bed net ownership and use, evening social activities, use of malaria preventive measures other than bed nets, perceived mosquito density and nuisance and malaria treatment.

From May until November in 2013, a structured observation survey was carried out. A first visit, in the evenings between 19.00 and 21.00 depending on the availability of the household, consisted of the observation of housing structures, people’s resting behaviour, bed net characteristics and topical repellent use of all household members. As actual bed net use at night could not be directly observed, bed nets that were suspended in the evenings before bedtime with at least two corners were considered ready for use. Holes in bed nets were observed but not systematically measured or counted. The next morning, a follow-up questionnaire was administered, exploring socio-economic status, seasonal sleeping spaces, perceived insect and mosquito protection, (alternative) use of nets, child care system, (alternative) repellent use, perceived mosquito nuisance, and previous malaria episodes was carried out with the household leader. Results regarding repellent use are not elicited in this paper.

#### Sampling

For the cross-sectional survey, 900 individuals were randomly selected from the MalaResT study population. In total, 393 individuals from 56 intervention villages and 431 from 57 control villages were located and answered the structured questionnaire.

For the structured observation survey in 2013, ten intervention villages and four control villages were purposively selected based on the criteria of malaria incidence. In each village, half of all households were randomly selected from the population census. Although there was no prior information available on which household had a farm or not, based on exploratory qualitative research, it was assumed that the majority of households did have a farm and commuted between farm- and village house, favouring the farmhouse during the rainy season and the village house during the dry season. As such, each selected household was assigned either to a farm list (meaning this household had to be observed and interviewed on their farm or rice field, if they had any) or to a village list (the interviewer had to observe and interview this household in their village house, if they had one) to explore potential differences between locations. A total of 653 households were randomly selected for the farm lists, of which 472 households were eligible (meaning they had a farm house where they stayed overnight); and 260 of those households were reached. A total of 655 households were randomly selected for the village lists, of which 555 were eligible (meaning they had a house in the village where they stayed overnight); and 291 households were reached. The main reason for not reaching households was because the selected family was not staying overnight at the respective location within the timeframe of the survey. Finally, a total of 431 households from the intervention arm and 120 households from the control were observed and interviewed.

For both surveys, an additional non-response form – recording reasons for not reaching the household - was used to measure possible systematic self-selection bias. The possible correlation between malaria infection and overnight stays at different locations was additionally tested in a malariometric survey in October 2012, on a sample of 6,640 individuals selected randomly out of the population censuses of the 113 villages included in the MalaResT study. Out of the list of 6,640 selected individuals, 4,996 individuals were reached.

### Entomological collections

To estimate human exposure to malaria vectors, pooled data of different entomological surveys conducted in Ratanakiri province are presented here. Prior to the MalaResT project, indoor and outdoor human landing collections were carried out in two villages (Phi and Lom) during four surveys of five to eight days in July-August 2009, July-November 2010, and July-August 2011. Human landing collections lasted from 18.00 until 06.00. Three collection points were chosen in each village, with paired indoor and outdoor collections per collection point. Within the MalaResT study only outdoor human landing collections were carried out in two intervention (Koy, Chrung) and two control (Kreh, Klis) villages selected out of 113 villages included in the project based on their malaria incidence, their accessibility and the availability of mosquito collectors. In every village, eight entomological surveys of ten successive nights were organized every two months between April and October of 2012 and 2013. Human landing collections lasted from 17.00 until 22.00 and from 17.00 until 08.00. Seven collection points were chosen per village in front of houses across the village, making a collection effort of 70 man-nights per village per survey. The same collection points were maintained throughout both studies. A rotation of collectors was ensured. Mosquitoes were identified and processed according to procedures described in Durnez *et al.* [13]. Mosquito collections were pooled per collection context and per collection hour for data visualisation. Results regarding changing mosquito behaviour in relation to repellent use are not elicited in this paper as they are subject of a separate paper.

## Data analysis

### Anthropological data

#### Qualitative data

Qualitative data collection and analysis were performed concurrently and data analysis was an iterative process. Preliminary data were intermittently analysed in the field, and preliminary results were then translated into the question guides for follow-up interviews. Initial results were continuously confirmed or refuted in the field, until saturation was reached. Data were analysed in NVivo 9 Qualitative Data Analysis software (QSR International Pty Ltd. Cardigan UK) by refining and categorizing themes grounded in the data.

#### Quantitative data

Preliminary analysis of the qualitative data was used to build the standardized questionnaires for the quantitative survey. The quantitative data was entered in Epi Info 7. The dataset was analysed in SPSS (IBM SPSS Statistics 19). Frequency tables for the main outcome variables were produced. Odds ratios and p-values for the association between overnight stays at the plot hut or in the forest and malaria infection were calculated using logistic regression models with a random intercept to adjust for clustering at village level.

### Entomological data

Mosquito collections were registered on standardized data collection forms, and data were entered in an Access database. Mosquito collections were pooled per collection context and per collection hour for data visualization. Boxplots indicating median biting times and 25 and 75 percentiles were constructed in R [25]. Exophagy was calculated as the proportion of mosquitoes biting outdoors as follows: O_18→06hrs_/(I_18→06hrs_ + O_18→06hrs_).

Human exposure to malaria vectors was estimated by the analysis of the following data: the weighting of the mean indoor and outdoor biting rates throughout the mosquito collection period by the proportion of humans that are typically indoors or outdoors at each time period, and this in the scenario of (i) no protective measures, and (ii) the observed use of protective measures. Each type of observed net being used was assigned a level of protection from exposure based on the levels of protection observed by Lines *et al.* in experimental hut trials of (i) untreated intact nets, (ii) untreated holed nets, (iii) intact permethrin-treated nets and (iv) holed permethrin-treated nets [26]. The proportion of indoor and outdoor mosquito bites averted by the use of protective measures was then calculated.

### Ethical considerations

The study protocol, including the anthropological, entomological and epidemiological parts, was approved by the National Ethics Committee for Health Research in Cambodia, the Ethics Committee of the University Hospital of Antwerp, and the Institutional Review Board of the Institute of Tropical Medicine of Antwerp. For the anthropological part, the interviewers followed the Code of Ethics of the American Anthropological Association (AAA). As proposed by the AAA, all interviewees were informed before the start of the interview about project goals, the topic and type of questions, the intended use of results for scientific publications as well as their right to reject being interviewed, to interrupt the conversation at any time, and to withdraw any given information during or after the interview. Anonymity was guaranteed and confidentiality of interviewees assured by assigning a unique code number to each informant. The interviewers sought oral consent from all interviewees. Oral consent was preferable, since the act of signing one’s name when providing certain information can be considered a potential reason for mistrust. Moreover, oral consent avoided the stress associated with potential illiteracy.

Regarding the entomological study, the mosquito collectors were informed about the objectives, process and procedures of the study and written informed consent was sought from them. Collector candidates were invited among the adult village population and if individuals wanted to withdraw they were allowed to do so at any time without negative consequences. Access to malaria diagnosis and treatment was guaranteed throughout the study. For the epidemiological study, survey participants or his/her parents or guardian provided informed written consent for individual participation.

## Results

### Variance in sleeping behaviour and bednet use in relation to vector biting times

#### Vector biting times

**Figure 1:**
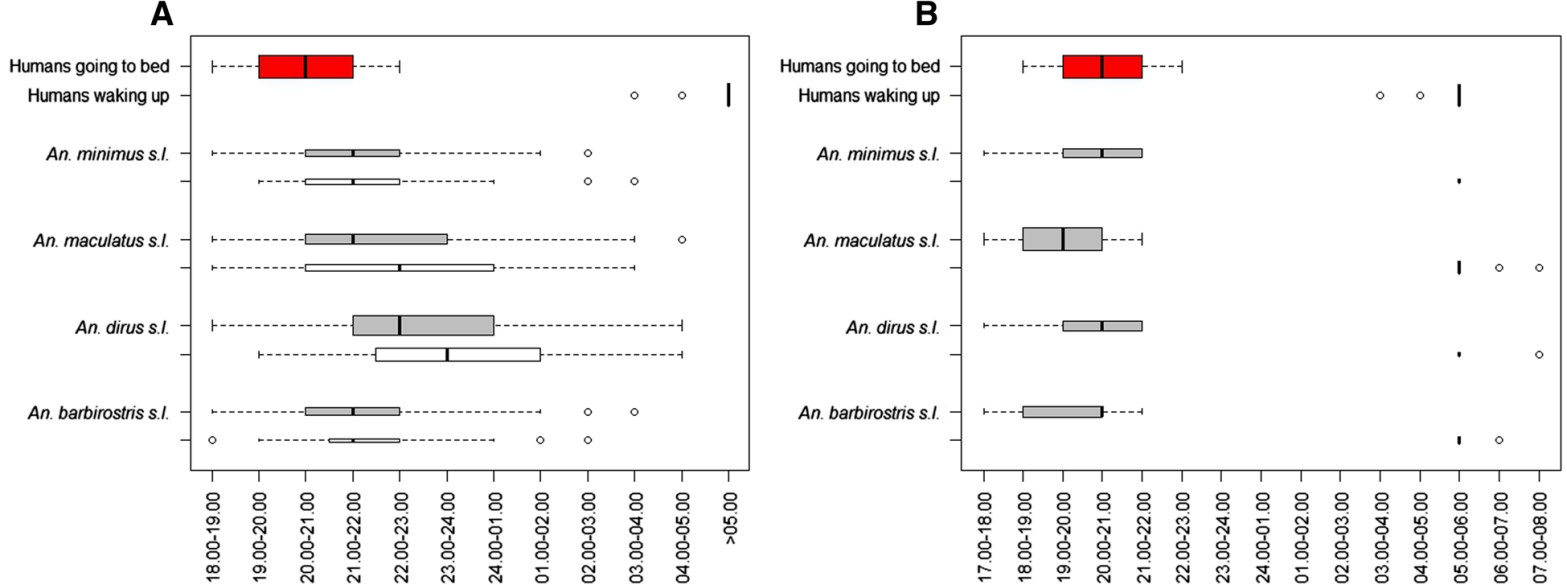
Local human sleeping and waking times (red box plots) compared to collection times of mosquitoes by human landing collections **A**. outdoors (grey boxplots) and indoors (white boxplots) in 2009–2011, and **B**. outdoors only during evening (17.00-22.00) and morning hours (05.00-08.00) in 2012–2013. No indoor or whole night collections were performed in 2012–2013. For both **A** and **B**, the size of the boxplot is proportional to the observed man biting rates in the respective study settings.

Human landing collection performed from 18.00 to 06.00 in 2009–2011 show that median biting times differ slightly between the known malaria vectors in Cambodia (*An. dirus s.l., An. minimus s.l., Anopheles maculatus s.l., Anopheles barbirostris s.l.*), and between outdoor and indoor collections (Figure 1A). *Anopheles dirus s.l.* has a median biting time of 22.00 - 23.00 outdoors and 23.00 - 00.00 indoors. The other vectors have a median biting time of 21.00 - 22.00 indoors and outdoors, except for *An. maculatus s.l.* with an indoor median biting time of 22.00 - 23.00. Outdoor mosquito collections organized only in the evening (17.00 - 22.00) and morning (05.00 - 08.00) in 2012–2013 showed that vectors can start biting from 05.00 onwards and continue to bite up to 08.00, although at very low biting rates (Figure 1B).

#### Human sleeping places and times

**Figure 2:**
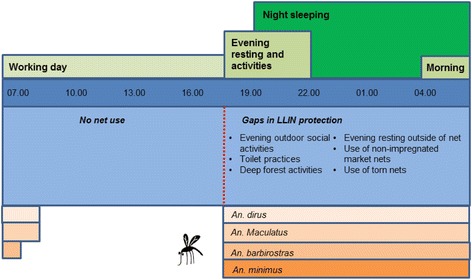
Suboptimal net use during vector biting times.

The place where people live and sleep varies seasonally. Throughout the year families alternate between sleeping in village homes (traditionally longhouses) and one or several homes at their farms in the forest, where dry rice and vegetables are grown (Table 1). The rainy season, which coincides with the malaria transmission season, is the most work intensive season for farmers, often leading to the preference to sleep at their forest farms (61.2%), while the dry season is usually spent in the village. This leads to variability in sleeping places directly related to malaria transmission, as spending the night at farms in the forest is a risk factor for malaria infection (OR 1.53, 95% CI 1.12 – 2.10, p < 0.01) (Table 2). Most respondents do report to keep separate bed nets at the farms (78.3%), however, rather than bringing nets back and forth from the village (20.4%) (Table 1). Observations during ethnographic research confirm that at farms, most people go to bed not long after sunset, exhausted from a long day’s field labour and with only minimal access to electricity. Around one quarter of respondents indicated they go to sleep before 19.00 when they are staying at their farms; about 70% of respondents do so between 19.00 and 21.00 and less than 10% after 21.00 (Table 3) (Figures 1A and 2). In villages, sleeping times are later than at forest farms and rice fields, as 31.9% of respondents reported to go to sleep after 21.00. In the mornings, around half of the respondents wake up before 06.00, both at farms and villages, when the main vectors are still active (Figure 1A).

**Table 1:** **Reported multiple residence system (Cross-sectional survey 2012)**

	**N**	**%**
Has farm(s)	768	93.2
Has a house on farm(s)	633	82.5
Sleeps at farm during malaria season	470	61.2
Has a bed net to use at farm	464	98.7
Brings back net back and forth from village	96	20.4
Keeps bed nets at farm	368	78.3
Has village house	755	91.6
Always sleeps in village during dry season	597	72.5
Always sleeps in village during rainy season	273	33.1

**Table 2:** **Association between overnight stays at plot hut or forest in past month and malaria infection**

	**Total**	**Positive**	**Malaria infection (all species)**	**OR**	**95% C.I.**	**p-value**
Plot hut overnight stay last month						
- Yes	2727	166	0.06	1.53	[1.12; 2.10]	<0.01
- No	2238	77	0.03			
Forest overnight stay last month						
- Yes	800	59	0.07	1.35	[0.97; 1.90]	0.08
- No	4165	184	0.04

**Table 3:** **Reported sleeping and waking times at the village and farm (Cross-sectional survey 2012)**

	**Village (n = 755)**		**Farm (n = 470)**	
	N	%	N	%
Sleeping times				
- Before 19.00	77	10.2	94	20.0
- Between 19.00 and 20.59	421	55.8	326	69.4
- At or after 21.00	241	31.9	44	9.4
- Missing	16	2.1	6	1.3
Waking times				
- Before 06.00	354	46.9	211	44.9
- At or after 6.00	384	50.9	254	54.0
- Missing	17	2.3	5	1.1

#### Use, state and perceived protection of different types of nets

Most respondents reported having a LLIN distributed by the national malaria control programme (98.5%) and sleeping in one (79.1%) (Table 4). 40.0% owned a bed net bought from the market, half of which reported to sleep in one. When observing households, in 5.3% of cases there were no indications of any net use; 45.4% were observed to be using (an) intact LLIN(s) and 23.2% intact non-impregnated market nets. In 24.7% of households (a) holed LLIN(s) was being used and in 16.5% (a) holed market net(s) (Table 5). The majority of household leaders stated mosquitoes (57.7%) and other small insects (64.1%) were able to enter the LLINs (Table 4). Only 14.8% of those household leaders thought the mosquito died from the insecticide after coming into contact with the LLIN. In contrast to LLINs, only a minority reported mosquitoes (17.0%) and small insects (20.0%) to be able to enter their market nets.

**Table 4:** **Reported bed net coverage and use**

**Cross-sectional survey 2012 (n = 824)**	**N**	**%**
Owns LLIN	812	98.5
Sleeps in LLIN	652	79.1
Owns market net	330	40.0
Sleeps in market net	162	19.7
Structured observation survey 2013 (n = 551)	N	%
Mosquitoes enter LLIN	318	57.7
Mosquitoes die when entering LLIN	47	14.8
Small insects enter LLIN	353	64.1
Small insects die when entering LLIN	75	21.2
Mosquitoes enter market net	45	17.0
Small insects enter market net	53	20.0
Which net do you use in the village? (n = 489)*		
- programme	343	70.1
- bought	136	27.8
- have no BN in village	10	2.0
Which net do you use at the farm? (n = 416)*		
- programme	336	80.8
- bought	65	15.6
- have no BN in farm	15	3.6
Children sleep without net while parents are still awake (n = 370)	244	65.9

*only those who report to also sleep at their farm/village house.

**Table 5:** **Observed bed net coverage and use (Structured Observation Survey 2013)**

	**Village**		**Farm**		**Total**	
	**n**	**%**	**n**	**%**	**n**	**%**
Observation bed nets						
HH observed not using nets	15	5.2	14	5.4	29	5.3
HH observed using LLIN(s)	187	67.8	176	71.5	363	69.5
HH observed using market nets	118	42.8	84	34.1	202	38.7
HH observed using intact LLIN(s)	138	47.4	112	43.1	250	45.4
HH observed using intact market nets	77	26.5	51	19.6	128	23.2
HH observed using torn LLINs	60	20.6	76	29.2	136	24.7
HH observed using torn market nets	53	18.2	38	14.6	91	16.5
HH observed having intact LLINs ready for use	129	44.3	106	40.8	235	42.6
HH observed having intact market nets ready for use	75	25.8	51	19.6	126	22.9
HH observed having torn LLINs ready for use	57	19.6	74	28.5	131	23.8
HH observed having torn market nets ready for use	53	18.2	37	14.2	90	16.3
Observation evening resting						
HH where children were observed sleeping	102	35.1	143	55.0	245	44.5
- Without net	76	74.5	105	73.4	181	73.9
- With net	26	25.5	38	26.6	64	26.1
HH where adolescents were observed sleeping	31	10.7	30	11.5	61	11.1
- Without net	24	77.4	22	73.3	46	75.4
- With net	7	22.6	8	26.7	15	24.6
HH where adults were observed sleeping	42	14.4	44	16.9	86	15.6
- Without net	30	71.4	36	81.8	66	76.7
- With net	12	28.6	8	18.2	20	23.3
HH where elderly were observed sleeping	26	8.9	18	6.9	44	8.0
- Without net	18	69.2	14	77.8	32	72.7
- With net	8	69.2	4	22.2	12	27.3
HH where somebody was observed sleeping (all age categories combined)	133	45.7	161	61.9	294	53.4
- Without net	107	80.5	130	80.7	237	80.6
- With net	26	19.5	31	19.3	57	19.4

#### Net preference

**Figure 3:**
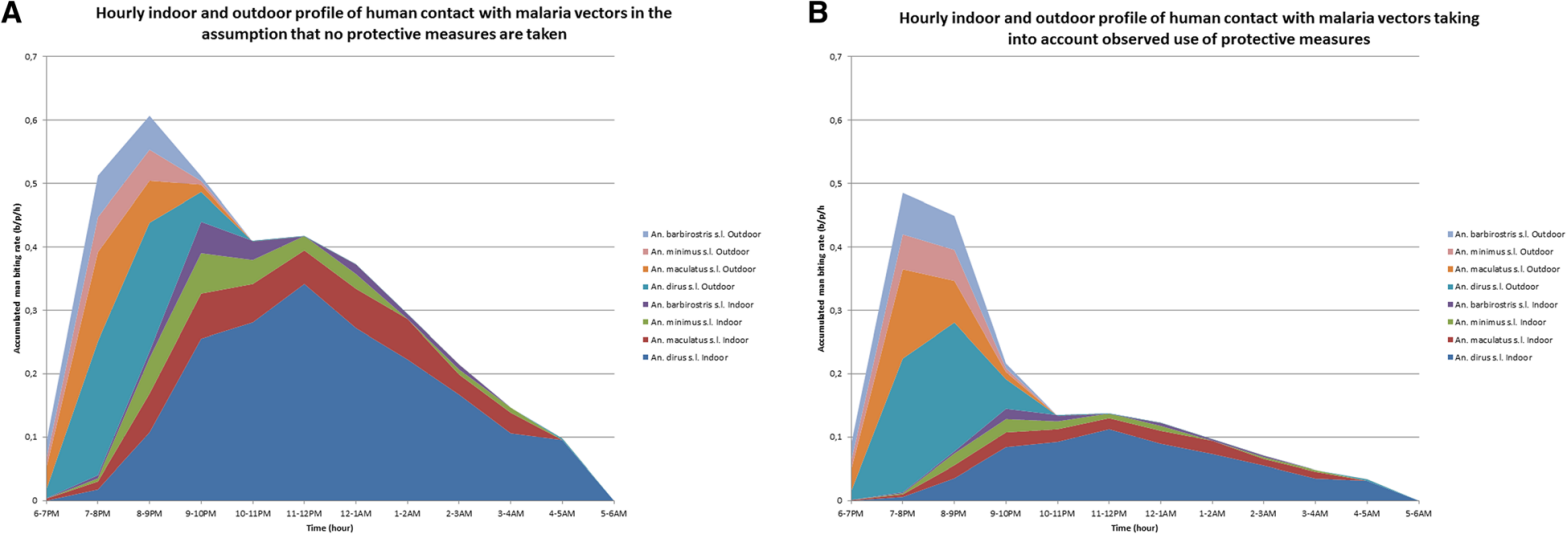
The hourly indoor and outdoor profile of human contact with malaria vectors in Ratanakiri province, Cambodia based on entomological data collected during 2009–2011 and human behaviour data collected during 2012–2013. The stacked line graph presents estimates of accumulated indoor and outdoor human contact rates with the four most common malaria vectors collected in the study area (*Anopheles dirus s.l*., *Anopheles minimus s.l., Anopheles maculatus s.l.,* and *Anopheles barbirostris s.l*.)*.* The movement pattern of people was taken into account by weighting the vector biting rates throughout the night by the proportion of humans that are typically indoors or outdoors at each time period. **(A)** No weighting by use of vector control tools; **(B)** Weighting by observed use of vector control tools.

Qualitative research showed that many people prefer market nets over LLINs, which are reported to be made of coarse fabric, considered too small to hold a large family, easy to break and to have such a big mesh size that mosquitoes and other insects can enter despite the insecticide. Moreover, the insecticide is perceived to stop working after a couple of weeks to one year. Consequently, many people own and use the colourful, soft and big nets that are bought non-impregnated from the market. The main perceived advantages of these market nets are the large size, accommodating larger families and preventing the net from creeping up, the colourful designs, and the small mesh size perceived to better prevent mosquitoes/insects from entering. The qualitative study indicated families to prefer to sleep together in the large-sized market nets when staying in their larger village homes; while the smaller LLINs were preferred for small farmhouses or bamboo constructions at the farm or rice field. When quantifying this variable during the structured observation survey, 24.6% reported to prefer market nets to the distributed LLINs (Table 4) (Figure 3).

### Variance in evening and outdoor activities in relation to vector biting places

#### Vector biting place

Between 2009 and 2011, the majority of malaria vectors were collected outdoors, which was 70% of all mosquito species collected. Slight differences in exophagy rates were observed between vector species (80% for *An. barbirostris s.l.*, 70% for *An.dirus s.l.*, 68% for *An. maculatus s.l.*, and 66% for *An. minimus s.l.)*. For *An. dirus s.l.* and *An. maculatus s.l.*, the median outdoor biting time was one hour earlier as compared to the median indoor biting time (Figure 1A).

#### Open housing structures

Completely or partially open houses (i.e. without exterior walls) and bamboo-thatched plot huts only provide minimal protection from insects during evening activities. More open and/or thatched roof houses were located at forest farms as compared to the villages (Table 6). Entomological data suggests that people are still exposed to ‘outdoor’ mosquito biting in the evenings while sitting inside their open houses (though less than outside) (Figure 1), and anthropological data indicates this is especially the case at farmhouses located in the forest where the malaria risk is higher.

**Table 6:** **Observed housing characteristics per location (Structured observation survey 2013)**

	**Village (n = 291)**		**Farm (n = 260)**	
	**N**	**%**	**N**	**%**
Stilted house	252	86.6	211	81.2
Roof				
- thatch	35	12.0	104	40.0
- tin	247	84.9	150	57.7
- tile	7	2.4	0	0
- plastic	2	0.7	6	2.3
Walls				
- plastic sheeting walls	37	12.7	75	28.8
- no walls or only partly walled	98	33.7	130	50.0
Permanently open windows	179	61.5	201	77.3

#### Social evening activities

During evening hours when vectors are active, many people engage in various in-house and outside activities (basket weaving, collecting water and firewood, tending to cattle, watching television, etc.). These activities vary according to the location (Table 7) due to differences in the availability of electricity. At the forest farms and rice fields, only a small number of families have a battery or a generator, allowing them to show movies on DVD in the evening, attracting mostly children from across other forest farms. In villages, more than half of the respondents reported to have some form of power to use in the evenings, resulting in considerably more people engaging in social activities in the evening, such as watching television (Table 8).

**Table 7:** **Reported evening activities at farms (Cross-sectional survey 2012)**

	**N**	**%**
Evening activities at farms (n = 470)		
Housework related activities (handicraft, cleaning, cooking, etc.)	318	67.7
Tending cattle	27	5.7
Nothing/chatting	181	38.5
Forest activities (gathering firewood, fishing, hunting, etc.)	48	10.2
Multimedia (tv)	43	9.1
Other (reading, bathing, having dinner)	33	7.0

**Table 8:** **Access to electricity in the village (Cross-sectional survey 2012)**

	**N**	**%**
Household’s access to power (n = 824)	511	62.0
Personal generator	175	21.2
Battery	352	42.7
Solar power	35	4.2
Large village generator	42	5.1
Power grid	1	0.1

People use smoke from fires or from cigarettes outdoors during these evening biting hours to decrease mosquito nuisance, especially when at farms or in the deep forest where mosquito nuisance is reportedly higher (Table 9). Although the majority of respondents reports to also protect themselves from mosquito biting by wearing long clothes, observation in villages shows that children usually have either lower or upper bodies completely exposed, and adults’ clothes are only partially covering the body (due to large rips or men wearing only long trousers).

**Table 9:** **Reported mosquito bite protective measures at different locations (Cross-sectional survey 2012)**

	**Village (n = 773)**		**Farm (n = 759)**		**Deep forest (n = 550)**	
Smoke from fire	337	43.6	476	62.7	112	20.4
Smoke from cigarettes	171	22.1	199	26.2	184	33.5
Coils	44	5.7	33	4.3	13	2.4
Clothes with long sleeves/pants	523	67.7	676	89.1	513	93.4
Insecticide sprays	36	4.7	27	3.6	3	0.5
Traditional methods (herbs, etc.)	2	0.3	2	0.3	3	0.5

#### Evening resting

Qualitative research showed that, more than adults, children often rest (and fall asleep) on the floor in the house or on bamboo beds or hammocks outside, and more so at forest farms than in the village (Figure 2). Such ‘resting behaviour’ is not considered the onset of the night’s sleep, and is mostly done without bed net protection, despite often occurring in ‘sleeping places’ and during prime vector biting time (Figures 1A and 2). It is only later in the evening, when all members of the household go to sleep and actual ‘night time’ sets in, that children are put inside the bed net with their parents. During the structured observation survey, at forest farms 55.0% of observed households had children taking a nap before actual sleeping time and doing so without bed nets in 73.4% of cases (for resting behaviour of other age categories, see Table 5). The majority of household leaders (65.9%) also report to let their children sleep unprotected while parents are still awake in the evening. When looking at all age categories combined, people were observed sleeping in the evenings in 53.4% of households, and doing so unprotected in 80.6% of cases (Table 5).

#### Deep forest economic activities

Most of the indigenous population (67.2%) engaged in economic forest activities (Table 10), of which 23.1% reported to also spend nights in the forests (Figure 2). Moreover, 52.0% of the forest-goers are engaged in fishing and hunting, which often happens during the night. There was a trend towards increased odds of malaria infection associated with spending a night in the deep forest in the last month, however, there was limited statistical evidence for this association (OR 1.35, 95% CI 0.97 – 1.90, p = 0.08) (Table 2).

**Table 10:** **Reported outdoor deep forest economic activities (Cross-sectional survey 201**

	**N**	**%**
Performs deep forest activities	554	67.2
Hunting	165	29.8
Fishing	236	42.6
Logging	151	27.3
Collecting forest products (bamboo shoots, fruit, honey, firewood, etc.)	477	86.1
Other	43	7.8
Stays overnight in the deep forest:		
- Never	418	75.5
- Often	3	0.5
- Sometimes	128	23.1

#### Toilet practices

Qualitative research showed that people urinate and defecate in the forest surrounding the village or the farm, and even call this “going to the forest” (Figure 2). There are few, if any, latrines, toilets or other sanitary constructions in the villages, and none at the forest farms and rice fields. People report to be very bothered by mosquito bites while going to the toilet, as usually several parts of the body become exposed. As people also go to the toilet during evening, night and morning hours, this activity potentially constitutes a risk for malaria infection.

### Exposure to malaria vector bites in relation to the use of vector control tools

After adjusting for the typical movement of people, and not taking into account the use of vector control tools, the greatest part of potential vector exposure still occurs indoors (73%) (Figure 3A). When taking into account the observed use of intact LLINs and market nets (assuming an 85% efficacy), holed market nets (assuming a 24% efficacy), and holed LLINs (assuming a 71% efficacy) [23] in the village only (entomological data from farms not available), the portion of indoor vector exposure still consisted of 47% of the total exposure (Figure 3B). Taking into account protection levels of LLINs and market nets being used and local sleeping times, in the study context, the observed use of all nets is estimated to decrease indoor exposure by 67%.

## Discussion

In low transmission settings, finding appropriate strategies to prevent residual malaria transmission, including transmission due to early and outdoor biting, is currently one of the major challenges in Southeast Asia [27,28]. A close look at the characteristics of the relationship between vector and human behaviour shows a complex interaction over time and place. The resulting heterogeneity is related to the presence of early and outdoor biting malaria vectors, the slash-and-burn farmers’ multiple residence system, locally used (partially) open housing structures, variance in labour and social activities, sleeping times according to the place of residence and season, and variance in bed net use depending on related user preferences.

Although heterogeneity of human and mosquito behaviour is of general importance to malaria control, and as such transcends contextual particularities, in-depth socio-cultural research is needed to explore the way local communities shape this heterogeneity. In the current study context, focusing on ethnic minorities socio-culturally different from the larger Khmer society in Cambodia, more transmission occurs at forest farms than in the villages, which has also been shown in similar Southeast Asian contexts where ethnic minorities rely on the forest for farming [9,18]. In addition, the indoor/outdoor distinction is less clear at forest farms as housing here is often completely or partly open and differs from corresponding village homes. As such, the malaria vectors, which in this context preferably bite outdoor, will not be entirely prevented from biting people inside these open houses, challenging the current conceptualization of outdoor transmission. Moreover, the fact that people commute between these different houses with different levels of exposure to indoor and outdoor mosquito biting, results in a constantly varying vulnerability to malaria. Reported sleeping times also vary according to the context. People at fields go to bed earlier than in the villages and earlier than expected as entomological early man biting proportions, (EBP) calculated in similar contexts in Southeast Asia, often take 22.00 as cut-off times for people going to sleep [13,27]. However, these calculations could be more accurate using locally researched sleeping times as this directly impacts on the proportion of infections that can be accounted for by early or nighttime biting.

In a previous study conducted in Vietnam, also approximately half (52%) of respondents were asleep by 19.00, only 24.5% were still awake after 20.00, and by 21.00 almost everybody (92%) was asleep. Here, sleeping times at farms, where most transmission occurred, were also consistently earlier than in villages and earlier than used for calculating EBP [9]. The same applies to the social evening activities varying according to the place and time of the year. Outdoor evening activities during early vector biting hours occur less during the agricultural peak season (which coincides with the malaria season), especially at those residences in the more forested areas where farmers are tired after an exhausting day’s work and the lack of electricity limits social activities. Although malaria vectors start biting outdoors as early as 17.00 and that they continue to do so until 08.00, for the majority of people that sleep under bed nets, the window for outdoor and early transmission was shorter than expected. These earlier than expected sleeping times in settings with a high bed net coverage could be responsible for further driving the selection of early-biting mosquitoes. Conversely, the small percentage of people going to sleep under bed nets after 21.00 or the people that do not use any bed nets or only torn market nets, could disproportionately contribute to malaria transmission. Without LLINs, exposure does indeed still occur largely indoors.

One of the defining elements of residual transmission is based on the premise of total LLIN coverage [1]. While survey results report high LLIN use, due to response bias and the often suboptimal operationalization of the concept ‘net use’ in questionnaires, actual LLIN use may not be as high as self-reported data from surveys indicate [5]. This is also suggested by the direct observation of the amount of households where market nets were being used, which corresponded to the percentage of people reporting *to own a market net* and not to the reported percentage of market net *use*, which was about 20% lower. In addition, many of these non-treated market nets were holed, offering only very limited protection [26], which, in turn would result in lower than expected community-level protection of non-users [29].

In addition to 30% of households not using LLINs, various gaps in protection were identified during vector biting times, which related, among others, to children and adults resting outside or inside in sleeping spaces in the evening before their reported sleeping times and, at night, when using non-treated torn nets while sleeping in often open housing. Additional gaps in night protection, however, cannot be addressed with LLIN, such as outdoor economic forest activities and toilet practices. Moreover, entomological data confirms that without LLIN, and not considering the potential protective mass-effect of LLIN in a village, a considerable proportion of exposure does indeed occur indoors. Considering all these factors, the contribution of night transmission may still be underestimated.

It has been hypothesized that controlling malaria with LLINs has certain fundamental limitations in regions characterized by early and outdoor biting, thus improving coverage of LLINs alone might not achieve malaria elimination [1]. It is clear that additional interventions aiming for personal protection during evening and night activities are essential. However, based on the current evidence, additional efforts in improving LLIN use during times when people are resting in the evening and during the night may still have an impact on further reducing malaria transmission in Cambodia.

Re-imagining malaria interventions by focusing not only on the heterogeneity in malaria transmission, but more specifically on the connection between heterogeneous human and vector behavior, is crucial when evaluating what works, what is still missing, and how to accelerate the progress in malaria control towards elimination.
